# Supplementary material for: Content-rich biological network constructed by mining PubMed abstracts
Source: BMC Bioinformatics. 2004 Oct 8;5:147. doi: 10.1186/1471-2105-5-147 (PMC528731; doi:10.1186/1471-2105-5-147)
Supplement: Additional File 2 — The original results of the above study (non-essential files are deleted to keep the file size under the limit set by BMC bioinformatics). [file 1471-2105-5-147-S2.bz2 › chilibotAdditionalFile2/dip20/140ID7739537E499/html/E2F2_RB1.html]

 


 **E2F2** and **RB1** 
  
Found 31 abstracts in PubMed, retrieved 20.  
 

 What does Google say? 
 PDF only 
| .edu only 

---

**Interactive relationship** (e.g. stimulation, inhibition, etc)

**Non-interactive relationship** (e.g. studied together, co-existance, homology, etc.)

- For E2F 1, E2F 2  [ **E2F2** ] , and E2F 3, the repressor is the product of the retinoblastoma  [ **RB1** ]  gene, pRb.  Ref: 7760804 Mol Cell Biol, 1995
- Consistent with previous studies, E2F 1, E2F 2  [ **E2F2** ] , and E2F 3 bind specifically to the retinoblastoma  [ **RB1** ]  protein.  Ref: 8657117 Mol Cell Biol, 1996
